# A new genus and species in the mite family Eupodidae (Acari, Eupodoidea) from Crimea

**DOI:** 10.3897/zookeys.422.7802

**Published:** 2014-06-30

**Authors:** Alexander A. Khaustov

**Affiliations:** 1 Tyumen State University, Tyumen, Russia

**Keywords:** Acarina, Eupodoidea, Eupodidae, *Pseudoeupodes*, systematics, key, Crimea

## Abstract

A new genus *Pseudoeupodes* Khaustov, **gen. n.** and new species *Pseudoeupodes porosus* **sp. n.** are described from moss in Crimea. The taxonomy of the Eupodidae and some other families and genera of Eupodoidea is reviewed. The genus *Turanopenthalodes* Barilo, 1988 is transferred from Penthalodidae to Penthaleidae. The family Cocceupodidae Jesionowska, 2010 and the genus *Filieupodes* Jesionowska, 2010 are considered as junior synonyms of Eupodidae Koch, 1842 and *Cocceupodes* Thor, 1934, respectively. A key to genera of the family Eupodidae is provided.

## Introduction

Mites of the cosmopolitan superfamily Eupodoidea Koch, 1842 are fungivorous, phytophagous and predatory. The classification of the superfamily lacks stability (Baker and Lindquist 2002). The superfamily Eupodoidea currently includes nine families: Eupodidae Koch, 1842, Penthaleidae Oudemans, 1931, Penthalodidae Thor, 1933, Rhagidiidae Oudemans, 1922, Strandtmanniidae Zacharda, 1979, Eriorhynchidae Qin & Halliday, 1997, Pentapalpidae Olivier & Theron, 2000, Dendrochaetidae Olivier, 2008 and Cocceupodidae Jesionowska, 2010 (Jesionowska 2010; Walter et al. 2009). The validity of the latter two families is problematic in my opinion (see Discussion). The family Eupodidae includes two subfamilies: Benoinyssinae Fain, 1958 and Eupodinae Koch, 1842, although this subdivision is not followed by most workers. The genera *Cocceupodes* Thor, 1934 and *Linopodes* Koch, 1835, which were previously placed in the family Eupodidae, were recently transferred to the separate family Cocceupodidae (Jesionowska 2010); and the genus *Hawaiieupodes* Strandtmann & Goff, 1978 was transferred to the family Penthalodidae (Jesionowska 2008).

This paper presents a description of a new genus and species of eupodid mite, *Pseudoeupodes porosus* gen. n., sp. n., collected from moss in Crimea, and discusses the taxonomy of some families and genera of Eupodoidea.

## Materials and methods

Mites were collected from moss using Berlese funnels and mounted in Hoyer’s medium. Notations for the prodorsal and leg setae follow Lindquist and Zacharda (1987) and Baker (1995), and the remaining nomenclature is as applied to eupodoids by Baker (1990). All measurements are given in micrometres (μm) for the holotype and for five paratypes (in parentheses). In descriptions of leg setation the number of solenidia is given in parentheses. Photographs were taken with a digital camera Tucsen 3.0 via the ocular of light microscope MBI-11 with phase contrast device. The type material is deposited in the mite collection of the Tyumen State University, Tyumen, Russia.

## Systematics

### Family Eupodidae Koch, 1842

#### Genus Pseudoeupodes

Taxon classificationAnimaliaTrombidiformesEupodidae

Khaustov gen. n.

http://zoobank.org/BABD612A-51F7-4111-A1D5-31760CE6208A

##### Type species.

*Pseudoeupodes porosus* Khaustov, sp. n. Monotypic.

##### Description.

**Figures 1–2.**
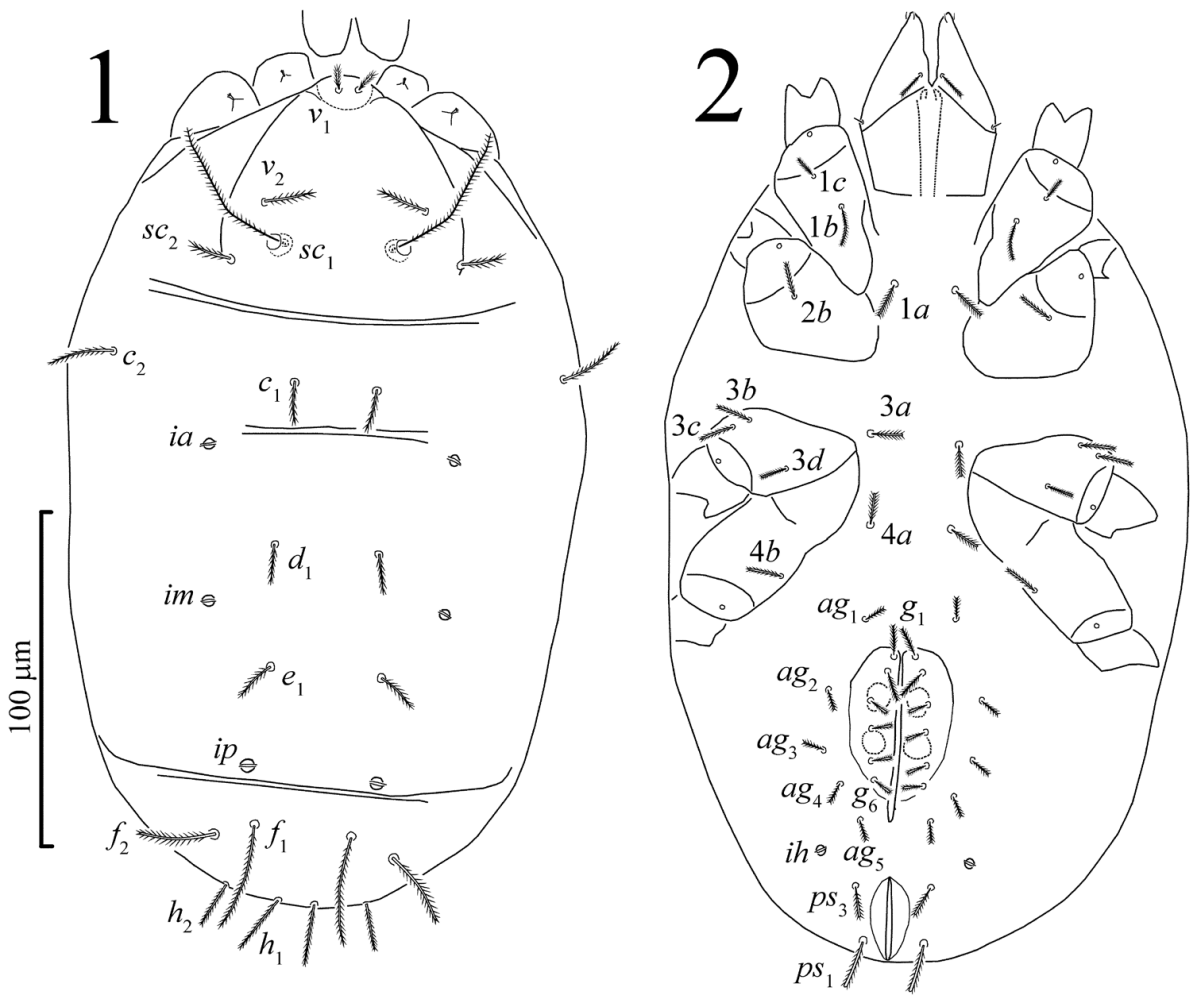
*Pseudoeupodes porosus* Khaustov, gen. n., sp. n., female: **1** idiosomal dorsum **2** idiosomal venter.

**Female.** *Idiosomal dorsum* (Figs 1, 11–13). Idiosoma oval. Cuticle soft and striated. Sejugal furrow well developed. Prodorsum with three pairs of tactile setae (*v*_1_, *v*_2_, *sc*_2_) and a pair of filiform trichobothria (*sc*_1_). Naso (epivertex) folded downward onto ventral surface of prodorsum, setae *v*_1_ situated on dorsal part of naso near anterior margin of prodorsum; naso defined by different pattern of striation from surrounding prodorsum (Fig. 11). Hysterosoma with eight pairs of dorsal setae (*c*_1_, *c*_2_, *d*_1_, *e*_1_, *f*_1_, *f*_2_, *h*_1_, *h*_2_) and three pairs of large round lyrifissures (*ia*, *im*, *ip*). Hysterosoma dorsally with two transverse furrows, between setae *c*_1_ and *d*_1_, and between *e*_1_ and *f*_1_. Setae *f*_1_ not trichobothrium-like.

**Figures 3–6. F2:**
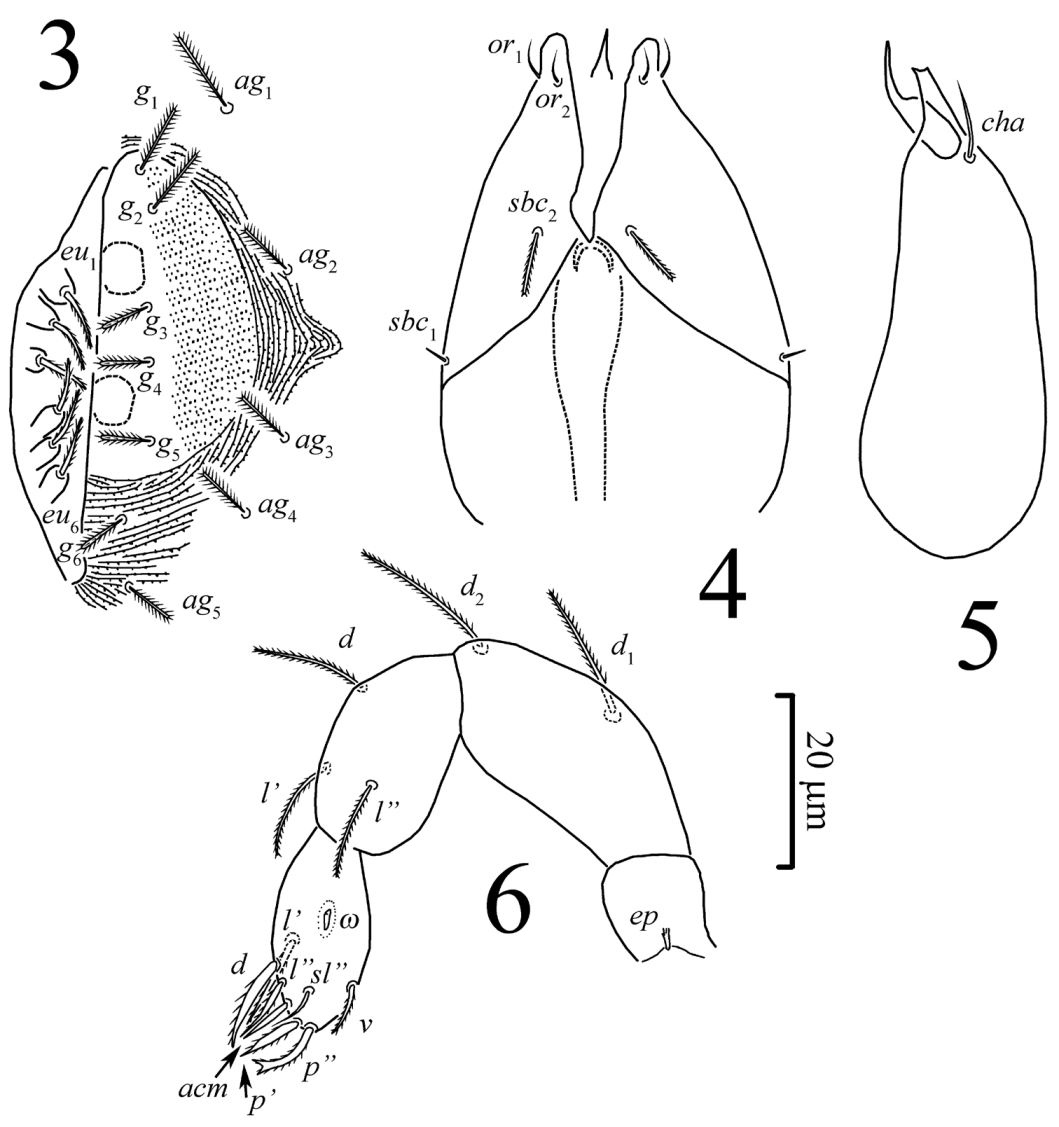
*Pseudoeupodes porosus* Khaustov, gen. n., sp. n., female: **3** genital area **4** subcapitulum **5** chelicera, antiaxial aspect **6** palp, antiaxial aspect.

*Idiosomal venter* (Figs 2, 3, 14–15). Coxisternal setal formula 3-1-4-2; six pairs of eugenital setae; six pairs of genital setae; five pairs of aggenital setae; two pairs of pseudanal setae; one pair of lyrifissures (*ih*), same form as dorsals.

*Gnathosoma* (Figs 4–6). Palp setal formula 0-2-3-8(ɷ), tarsus ovoid. Chelicerae: typical for eupodid mites, movable digit slender and acuminate distally, fixed digit distinctly shorter than movable digit and truncated distally; seta *cha* present.

**Figures 7–10. F3:**
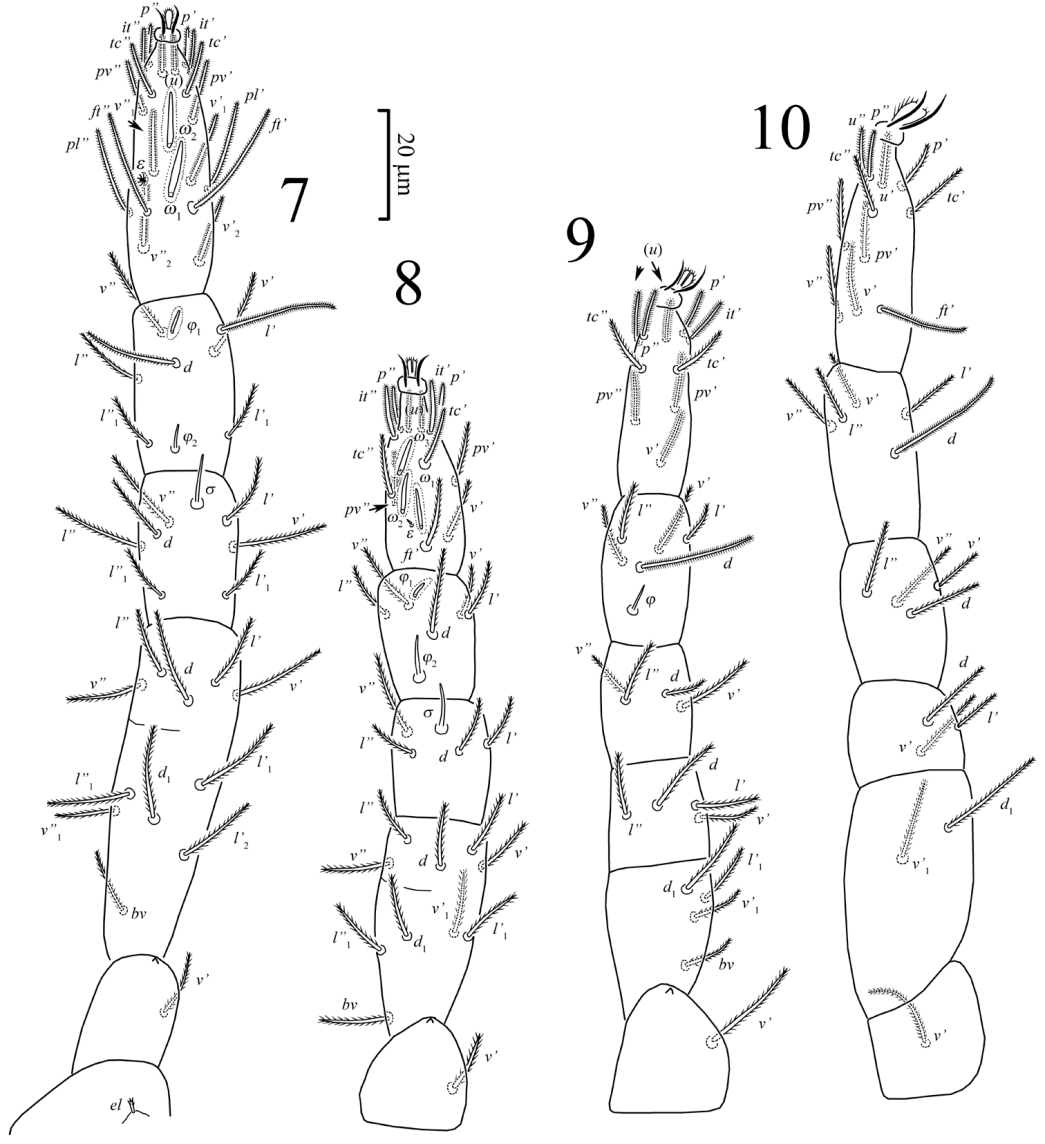
*Pseudoeupodes porosus* Khaustov, gen. n., sp. n., female: **7–10** legs I-IV, respectively.

*Legs* (Figs 7–10). All legs shorter than body. Soft cuticle separating coxisternal plates and trochanters of all legs with distinct pore-like structure (Fig. 14). Rhagidial organ I with two longitudinally arranged solenidia; rhagidial organ II with three longitudinally arranged rhagidial solenidia. Tarsus I with famulus (stellate setae) situated in shallow depression; tarsus II with spine-like famulus. Tibiae I and II with one distal rhagidial solenidion; tibiae I-III with proximal erect solenidion; genua I and II with one erect solenidion. Femur IV not enlarged. Trochanteral setal formula 1-1-1-1.

**Figures 11–15.**
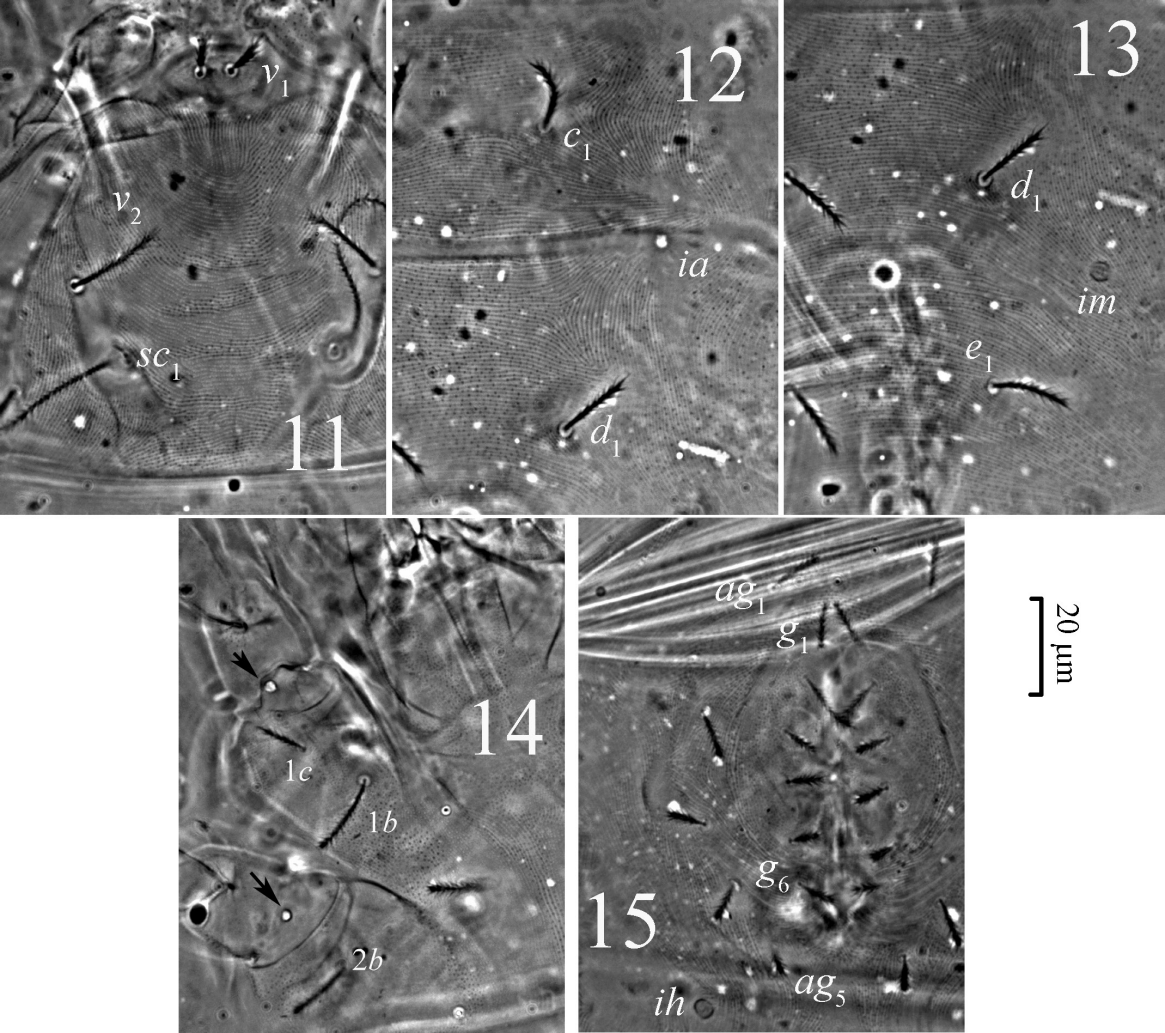
*Pseudoeupodes porosus* Khaustov, gen. n., sp. n., female: **11** prodorsum **12** striation in the area of setae *c*_1_ and *d*_1_ **13** striation in the area of setae *d*_1_ and *e*_1_ **14** venter of propodosoma, arrows point to pore-like structures **15** genital area.

**Male and immatures.** Unknown.

##### Etymology.

The genus name is derived from the related genus *Eupodes* and prefixed pseudo- (from Greek ψευδής) meaning false.

##### Differentiation of the genus.

The new genus is most similar to *Caleupodes* Baker, 1987. Both genera are characterized by the naso folded down to the ventral surface of the prodorsum, the same palpal chaetotaxy, six pairs of eugenital and five pairs of aggenital setae, the presence of only two pairs of pseudanal setae, femur IV not enlarged, trochanteral setal formula 1–1–1–1, and legs I-IV shorter than idiosoma. The new genus differs from *Caleupodes* by having striated dorsal cuticle (reticulated in *Caleupodes*), the absence of a transverse furrow between segments D and E (all hysterosomal segments are clearly separated by transverse furrows in *Caleupodes*), coxisternal setal formula 3–1–4–2 (3–1–4–3 in *Caleupodes*), and genua I and II with a solenidion (absent in *Caleupodes*). The new genus is also closely related to *Niveupodes* Barilo, 1991 in the naso folded down to the ventral surface of the prodorsum, femur IV not enlarged, trochanteral setal formula 1–1–1–1, legs I-IV shorter than idiosoma, and only two pairs of pseudanal setae. It differs from *Niveupodes* by the presence of dorsal transverse furrows between segments C – D and E – F (dorsal hysterosoma without transverse furrows in *Niveupodes*), coxisternal setal formula 3–1–4–2 (3–1–4–3 in *Niveupodes*), five pairs of aggenital and six pairs of eugenital setae (four aggenital and five eugenital in *Niveupodes*), the absence of scapular lyrifissure *isc* (present in *Niveupodes*, according to Barilo 1991), and the ovoid palptarsus (cone-shaped in *Niveupodes*). The new genus differs from all other known eupodoid genera by the presence of pore-like structures of unknown origin and function situated on the soft cuticle between the coxisternal plates and the trochanters of all legs. These pore-like structures are unknown in any other group of trombidiform mites.

#### Pseudoeupodes porosus

Taxon classificationAnimaliaTrombidiformesEupodidae

Khaustov sp. n.

http://zoobank.org/3897531B-758D-4AC5-B0F0-F9D85048964C

Figs 1 [Fig F2] [Fig F3] –15

##### Description.

**Female.** Length of idiosoma 288 (280–300), width 163 (157–180).

*Idiosomal dorsum* (Figs 1, 11–13). Idiosoma with striae bearing microtubercles (Figs 11–13). All dorsal setae densely pilose, setae *v*_1_ slightly widening apically. Prodorsum with pair of longitudinal lines between the bases of setae *sc*_2_ and anterior margin of prodorsum near setae *v*_1_. Setae *sc*_1_ with large barbs, a weak reticulate subcuticular ornamentation visible posteriorly to bases of *sc*_1_ (Fig. 11). Lyrifissures *ia* situated posterolaterally to bases of setae *c*_1_; *im* situated posterolaterally to bases of setae *d*_1_; *ip* situated between setae *e*_1_ and *f*_1_. Length of dorsal setae: *v*_1_ 9 (98-10), *v*_2_ 16 (15–19), *sc*_1_ 50 (47–55), *sc*_2_ 15 (14–17), *c*_1_ 13 (12–16), *c*_2_ 21 (19–25), *d*_1_ 16 (14–19), *e*_1_ 15 (14–18), *f*_1_ 40 (37–45), *f*_2_ 25 (24–28), *h*_1_ 23 (21–26), *h*_2_ 17 (15–21). Setae *f*_1_ longest of dorsal hysterosomal setae but not of trichobothrial form.

*Idiosomal venter* (Figs 2, 3, 14–15). All ventral setae densely pilose. Setae 1*a*, 3*a*, 4*a* (10–13) slightly widened distally; setae 1*c* the shortest on coxal fields. Genital setae arranged in one longitudinal row, anterior five pairs (*g*_1_–*g*_5_) situated on non-striated genital covers bearing only microtubercles, posterior pair (*g*_6_) situated outside genital covers on striated cuticle (Figs 3, 15). Anterior two pairs of genital setae distinctly longer (10–11) than other genitals (6–7). Aggenital setae increasing in length from *ag*_5_ (7) to *ag*_1_ (10–11). Eugenital setae situated on protuberances and arranged in three groups: two anterior (*eu*_1_, *eu*_2_), one medial (*eu*_3_) and three posterior (*eu*_4_-*eu*_6_) (Fig. 3). Pseudanal setae *ps*_1_ 15 (14–18) distinctly longer than *ps*_3_ 11 (10–14). Lyrifissure *ih* located anterolaterally to bases of setae *ps*_3_.

*Gnathosoma* (Figs 4–6). Integument papillate. Subcapitulum (Fig. 4) roughly triangular, with two pairs of minute smooth adoral setae (*or*_1_, *or*_2_), located subapically; subcapitular setae *sbc*_2_ densely pilose, *sbc*_1_ smooth, located laterally at level of proximal margin of palp trochanters, about one-third as long as *sbc*_2_, *sbc*_2_ inserted ventrally one third to one quarter of distance between *sbc*_1_ and tip of subcapitulum, labrum acuminate. Chelicera (Fig. 5) 60 in length. Palps (Fig. 6) with supracoxal seta *ep* minute, brush-like, femorogenual and tibial setae densely pilose, tarsal setae *acm* and *sl*” smooth, other tarsal setae pilose, *p*” bifurcate distally.

*Legs* (Figs 7–10). Relative lengths of legs: I>IV>III>II. All leg setae densely pilose. Leg I (Fig. 7): Supracoxal setae *ep* of the same shape and length as palpal supracoxal setae *ep.* Femur incompletely divided into basi- and telofemur. Setal formula: Tr – 1, Fe – 6+5, Ge – 7(1σ), Ti – 7(2φ), Ta – 18 (2ω, 1ε). Famulus ε (stellate seta) located in a shallow depression clearly posterolaterally to basal part of rhagidial solenidion ω_1_ and anterior to seta *ft*”. Rhagidial solenidion φ_1_ obliquely oriented toward anterior lateral margin of leg, located anterodorsally and situated in shallow depression; solenidion φ_2_ located posterodorsally in the space between setae *l*’_1_ and *l*”_1_; solenidion σ located anterodorsally, about 1.5 times as long as φ_2_. All setae on tarsus, *d* and *l*’ on tibiae are eupathidia (as applied for Eupodoidea by Lindquist and Zacharda 1987). Leg II (Fig. 8): Femur incompletely divided into basi- and telofemur. Setal formula: Tr – 1, Fe – 5+5, Ge – 4(1σ), Ti – 5(2φ), Ta – 12 (3ω, 1ε). Famulus ε spine-like, located just posterolaterally to base of rhagidial solenidion ω_1_. Rhagidial solenidion φ_1_ obliquely oriented toward anterior lateral margin of leg, located anterodorsally and situated in shallow depression; solenidion φ_2_ located posterodorsally; solenidion σ located anterodorsally, subequal to φ_2_. Tarsal setae (*u*), (*p*), (*it*) and *tc*’ are eupathidia. Leg III (Fig. 9): Setal formula: Tr – 1, Fe – 4+4, Ge – 4, Ti – 5(1φ), Ta – 10; femur completely divided into basi- and telofemur; solenidion φ located posterodorsally; all setae on tarsus and *d* on tibia are eupathidia. Leg IV (Fig. 10): Setal formula: Tr – 1, Fe – 2+3, Ge – 4, Ti – 5, Ta – 11; femur completely divided into basi- and telofemur; tarsal setae (*u*), *p*’, *ft*’ and *d* on tibia are eupathidia.

##### Type material.

Holotype female, slide № AK210494, **CRIMEA:** Yalta mountain-forest Nature Reserve, moss on soil, 21 April 1994, coll. A.A. Khaustov. Paratypes: five females, same data as holotype; seven female paratypes, **CRIMEA:** Yalta, moss on soil, 5 March 1994, coll. A.A. Khaustov.

##### Etymology.

The name of the new species refers to the presence of unusual pore-like structures in the soft cuticle between the coxisternal plates and the trochanters of all legs.

## Discussion

The present systematic organisation of the family Eupodidae and the superfamily Eupodoidea is highly unstable. Preliminary morphological cladistic analyses (Qin 1996, Qin and Halliday 1997) suggested that only two families (the Eriorhynchidae and Penthalodidae) are monophyletic. Of the other four, the Rhagidiidae plus Strandtmanniidae formed a monophyletic group, the Penthaleidae was paraphyletic, and the status of the Eupodidae was not resolved (Baker and Lindquist 2002). Jesionowska (1989) described the genus *Protopenthalodes* Jesionowska, 1989 in the family Penthalodidae and moved *Hawaiieupodes* Strandtmann & Goff, 1978 from Eupodidae to Penthalodidae (Jesionowska 2008). According to Jesionowska (2008), this new concept of the Penthalodidae will be published in a separate paper. Qin (1997) reconsidered the taxonomic position of *Protopenthalodes* and suggested that this genus is more appropriately placed in the family Eupodidae because of soft body integument. Barilo (1988) described *Turanopenthalodes* Barilo, 1988, which is another problematic genus in the family Penthalodidae. The key characters of the family Penthalodidae are the fully sclerotized body and the presence of an epirostrum projecting over the gnathosoma (Qin and Halliday 1997; Olivier 2008; Walter et al. 2009), but other characters such as the idiosomal setation have never been used to separate Penthalodidae from other families of Eupodoidea. In my opinion the genera *Hawaiieupodes* and *Protopenthalodes* are more closely related to Penthalodidae than to Eupodidae, in agreement with the suggestion by Jesionowska (2008). Like *Penthalodes* Murray, 1877, the type genus of the family Penthalodidae, both *Hawaiieupodes* and *Protopenthalodes* have the following synapomorphies: sejugal furrow not developed, lens-like eyes present near the setae *sc*_2_, setae *h*_2_ absent, naso usually very small, oval with minute setae *v*_1_. This combination of characters is not found in the closely related families Penthaleidae and Eupodidae, and following Jesionowska (2008), I retain *Hawaiieupodes* and *Protopenthalodes* in the Penthalodidae. The position of the genus *Turanopenthalodes* in the family Penthalodidae is doubtful. Barilo (1988) placed *Turanopenthalodes* in Penthalodidae based on a single character, the presence of epirostral processes lateral to the naso, similar to those found in the penthalodid genus *Stereotydeus* Berlese, 1901, which he considered the main differential character of the family Penthalodidae. Other apomorphic characters of this genus, such as neotrichy of the idiosoma, a small anal opening situated dorsally, short and truncated palptarsus and very characteristic “trident” at the distal end of the fixed digit of the chelicera, are similar to those found in *Penthaleus* Dugès, 1834, the type genus of the family Penthaleidae, and I currently place *Turanopenthalodes* in the family Penthaleidae. Undoubtedly some characters that are now used to separate some families in the Eupodoidea should be re-evaluated. Such an attribute as more conspicuously sclerotized dorsal body surfaces, which is characteristic of the family Penthalodidae (Walter et al. 2009), is highly variable. There is an undescribed species of *Protopenthalodes* in my collection with a soft body, but having subcuticular reticulate ornamentation throughout the body surface, which I consider as intermediate in the extent of body sclerotization.

The monotypic family Dendrochaetidae (Olivier 2008, 2009), which includes only the genus *Dendrochaetus* Olivier, 2009, shares some synapomorphic characters with *Hawaiieupodes* and *Protopenthalodes*. All these genera lack setae *h*_2_, the sejugal furrow is absent, the naso is small, almost round, and well separated from the anterior margin of the prodorsum. The only difference between Dendrochaetidae and soft-bodied Penthalodidae (*Hawaiieupodes* and *Protopenthalodes*) is the presence of an additional transverse furrow at the level of setae *v*_1_. *Dendrochaetes acarus* (Olivier, 2008) needs to be restudied and redescribed to clarify the status of the family Dendrochaetidae.

Jesionowska (2010) erected the family Cocceupodidae, in which she included three genera: *Cocceupodes* Thor, 1934, *Linopodes* Koch, 1835 and *Filieupodes* Jesionowska, 2010. According to Jesionowska (2010) the family Cocceupodidae differs from Eupodidae by two main characters: setae *v*_1_ situated posterior to naso and the presence of only two pairs of circumanal setae (*ps*_1_ and *ps*_3_). In my opinion, the decision to create the family Cocceupodidae is groundless. The similar location of setae *v*_1_ on dorsal part of naso near anterior margin of prodorsum is also found in the eupodid genera *Caleupodes*, *Niveupodes* and *Pseudoeupodes*, but in these genera the naso is not directed anteriorly, but folded to the ventral side of the prodorsum. The presence of only two pairs of pseudanal setae (*ps*_2_ absent, according to Baker 1990) is a variable character in the family Eupodidae. Setae *ps*_2_ are absent in the genera *Caleupodes*, *Niveupodes*, *Pseudoeupodes* and *Benoinyssus* Fain, 1958. The absence of setae *ps*_2_ is a reduction and could happen independently in different lineages of eupodoid mites (homoplasy). On the other hand, Jesionowska (2010) did not mention some synapomorphic characters of *Cocceupodes* and *Eupodes* Koch, 1842. Both genera have characteristic swollen femora IV adapted for jumping. Another synapomorphic attribute is the relatively long and thin legs I, which are usually subequal to or longer than the idiosoma, and much longer than legs II. In early derivative genera of Eupodidae, such as *Neoprotereunetes* Fain & Camerik, 1994, *Caleupodes* and *Niveupodes*, femora IV are not swollen and legs I are not so long and thin. Thus, the characters separating Cocceupodidae and Eupodidae proposed by Jesionowska (2010) are variable within the family Eupodidae. I therefore consider the family Cocceupodidae as a junior synonym of Eupodidae.

Jesionowska (2010) created a new genus *Filieupodes*, which differs from *Cocceupodes* by a single character, the filiform setae *v*_1_ (clavate or capitate in *Cocceupodes*). Filiform setae *v*_1_ is a plesiomorphic character state and should not be used for recognition of a new taxon. I therefore consider *Filieupodes* as a junior synonym of *Cocceupodes*.

Currently I recognize 11 genera in the family Eupodidae: *Xerophiles* Jesionowska, 2003, *Benoinyssus*, *Claveupodes* Strandtmann & Prasse, 1976, *Eupodes*, *Aethosolenia* Baker and Lindquist, 2002, *Neoprotereunetes*, *Linopodes*, *Cocceupodes*, *Niveupodes*, *Caleupodes*, and *Pseudoeupodes* gen. n.

### Key to the genera of the family Eupodidae

**Table:** 

1	Setae *f*_1_ trichobothrium-like	2
–	Setae *f*_1_ not trichobothrium	3
2	Setae *ps*_2_ present	*Xerophiles*
–	Setae *ps*_2_ absent	*Benoinyssus*
3	Setae *ps*_2_ present	4
–	Setae *ps*_2_ absent	7
4	Trichobothria (*sc*_1_) filiform	5
–	Trichobothria (*sc*_1_) clavate	*Claveupodes*
5	Femur IV not swollen	6
–	Femur IV swollen, adapted for jumping	*Eupodes*
6	Setae *h*_1_ trichobothrium-like, adanal setae present, tibia and tarsus I much thicker than other leg segments	*Aethosolenia*
–	Setae *h*_1_ not trichobothrium, adanal setae absent, tibia and tarsus I not enlarged	*Neoprotereunetes*
7	Leg I shorter or slightly longer than idiosoma, solenidia in rhagidial organs not T-shaped	8
–	Leg I more than 3 times longer than idiosoma, solenidia in rhagidial organs T-shaped	*Linopodes*
8	Leg I distinctly shorter than idiosoma, sejugal furrow well developed, femur IV not enlarged, naso folded to ventral surface of prodorsum	9
–	Leg I usually longer than idiosoma, sejugal furrow absent or poorly developed, femur IV enlarged, naso directed anteriorly	*Cocceupodes*
9	Hysterosoma dorsally with 3 pairs of lyrifissures	10
–	Hysterosoma dorsally with 4 pairs of lyrifissures (scapular lyrifissure present)	*Niveupodes*
10	Idiosoma dorsally reticulated, all hysterosomal segments delineated by distinct transverse furrows, solenidia on genua I and II absent	*Caleupodes*
-	Idiosoma dorsally striated, transverse furrows present only between segments C–D and E–F, solenidia on genua I and II present	*Pseudoeupodes* gen. n.

## Supplementary Material

XML Treatment for Pseudoeupodes

XML Treatment for Pseudoeupodes porosus
